# Hepatic transcriptome analysis provides new insights into ghrelin regulation of the liver in Nile tilapia (*Oreochromis niloticus*)

**DOI:** 10.3389/fvets.2023.1192195

**Published:** 2023-06-14

**Authors:** Huan Zhong, Chenyi Lou, Bingxin Ren, Jiamin Pi, Tao Dai, Weiling Qin, Yi Zhou

**Affiliations:** State Key Laboratory of Developmental Biology of Freshwater Fish, Life Science College, Hunan Normal University, Changsha, China

**Keywords:** ghrelin, liver, transcriptome, tilapia, growth

## Abstract

Ghrelin is a growth-promoting hormone produced by the gastrointestinal tract that plays a crucial role through the ghrelin-growth hormone secretagogue receptor (GHS-R) and growth hormone/insulin-like growth factor-1 (GH/IGF-1) axes. To explore the effect of ghrelin on the transcriptomic profile of tilapia liver, the hepatic transcriptome of tilapia was sequenced for two groups, including saline-injected control (CL) and ghrelin-injected (GL; 2 μg/g body weight) tilapia. The transcriptome of livers from the two groups was sequenced using an Illumina HiSeqTM 2000 platform and yielded approximately 310.53 million raw reads. Subsequently, approximately 308.51 million clean reads were obtained from the total raw reads using in-house Perl scripts. Approximately 92.36% clean reads were mapped to the Nile tilapia genome using RSEM. Using the DESeq package, 250 differentially expressed genes (DEGs) were identified. Kyoto Encyclopedia of Genes and Genomes (KEGG) analysis showed enrichment of two pathways related to RNA transcription (ribosome biogenesis in eukaryotes pathway and RNA transport pathway), with a total of 14 functional DEGs. ATP-binding and muscle contraction terms were identified as enriched using Gene Ontology (GO), yielding a total of 28 DEGs. Finally, real-time quantitative PCR (RT-qPCR) was used to confirm the accuracy of the transcriptomic results. The results of RT-qPCR were highly consistent with the RNA-seq, indicating that results of RNA-seq were valid. The differences in gene expression between the groups indicated that ghrelin-injection altered energy metabolism and RNA transcription in the tilapia liver, which provides new information to help promote the growth of tilapia.

## 1. Introduction

Nile tilapia (*Oreochromis niloticus*) is a fish of economic importance with a high nutritional value and tender meat (1, 2). Because of its characteristics of fast growth rate, omnivore diet, high resistance to low oxygen concentration and easy management for farming, Nile tilapia becomes the popular farmed fish over the world. Owing to the recent increase in the demand for high-quality protein, tilapia has been increasingly favored and the market demand for it has been expanding (3). Promoting the growth rate of tilapia and improving its quality have become issues of practical importance that can not only boost the supply of tilapia but also bring extensive economic benefits.

Liver is an important metabolic organ (4) in fish. Ingested food is digested and absorbed by the digestive system, and liver plays a crucial role in energy metabolism of nutrients. In liver, complex biochemical reactions occur, such as glycogen synthesis and glycogenolysis, lipid metabolism, and protein synthesis, which supply substances required in other organs and tissues (5). Furthermore, the liver produces a variety of hormones to regulate several biological processes. For example, it produces insulin-like growth factor-1 (IGF-1), which is a crucial growth-promoting hormone. IGF-1 acts on a variety of tissues, promotes cell division and differentiation, and stimulates protein synthesis (6). A previous study showed that the upregulation of IGF-1 promoted the growth of tilapia (7). Thus, the metabolic activity of the liver is closely related to the growth of the organism, and the changes in important genes in the liver are related to growth rates.

Importantly, the liver not only produces hormones, but it is also regulated by hormones (8). Ghrelin is a multifunctional hormone. In addition to regulating appetite and controlling insulin levels, it can also promote growth through the ghrelin-growth hormone secretagogue receptor (GHS-R) and growth hormone/insulin-like growth factor-1 (GH/IGF-1) axes (9). IGF-1 is mostly synthesized by hepatocytes; therefore, ghrelin may affect the liver through the ghrelin-GHS-R and GH/IGF-1 axes. A previous study has shown that ghrelin can alter the expression of immune-related genes in the liver of tilapia (10). In another study, two forms of ghrelin [n-decanoic (ghrelin-C10) and n-octanoic (ghrelin-C8)] were stored in micro-optical pumps. These pumps were implanted into experimental tilapia through abdominal surgery, and ghrelin was released at a rate of 10 ng/h. After 21 days, the experiment was terminated. The result showed that ghrelin-C10 had no effect on IGF-1 level in liver; however, it decreased plasma IGF-1 level and increased liver weight and lipid content. Ghrelin-C8 did not have a similar effect (11). These results provide evidence that ghrelin can regulate the expression of key genes in the liver. Moreover, peripheral ghrelin promotes fat deposition and gluconeogenesis in the liver of mouse (12). In the liver, ghrelin has been shown to inhibit the activity of adenosine 5′-monophosphate-activated protein kinase (AMPK) and the activation of this enzyme stimulated catabolism to supply ATP (13). In conclusion, ghrelin not only regulates hepatic activity through the ghrelin-GHS-R and GH/IGF-1 axes, but also acts directly on liver metabolism.

Liver is a major metabolic organ, and ghrelin is a pleiotropic growth-stimulating hormone. Clarifying the relationship between ghrelin and the liver and effects of ghrelin on the liver can promote the understanding of the growth mechanism of tilapia. To date, the effect of ghrelin on the transcriptome of tilapia liver has not been investigated. To determine liver functions and genes affected by ghrelin, we tested the transcriptome of tilapia injected with ghrelin and compared it with saline-injected tilapia. By comparing the differences in gene expression, differentially expressed genes (DEGs) were identified. Kyoto Encyclopedia of Genes and Genomes (KEGG) and Gene Ontology (GO) were used to identify the enriched pathways and GO terms to help understand the various roles of ghrelin and provide new information for the rational use of ghrelin to regulate hepatic gene expression of tilapia.

## 2. Materials and methods

### 2.1. Fish and injection

**Figure 1:**
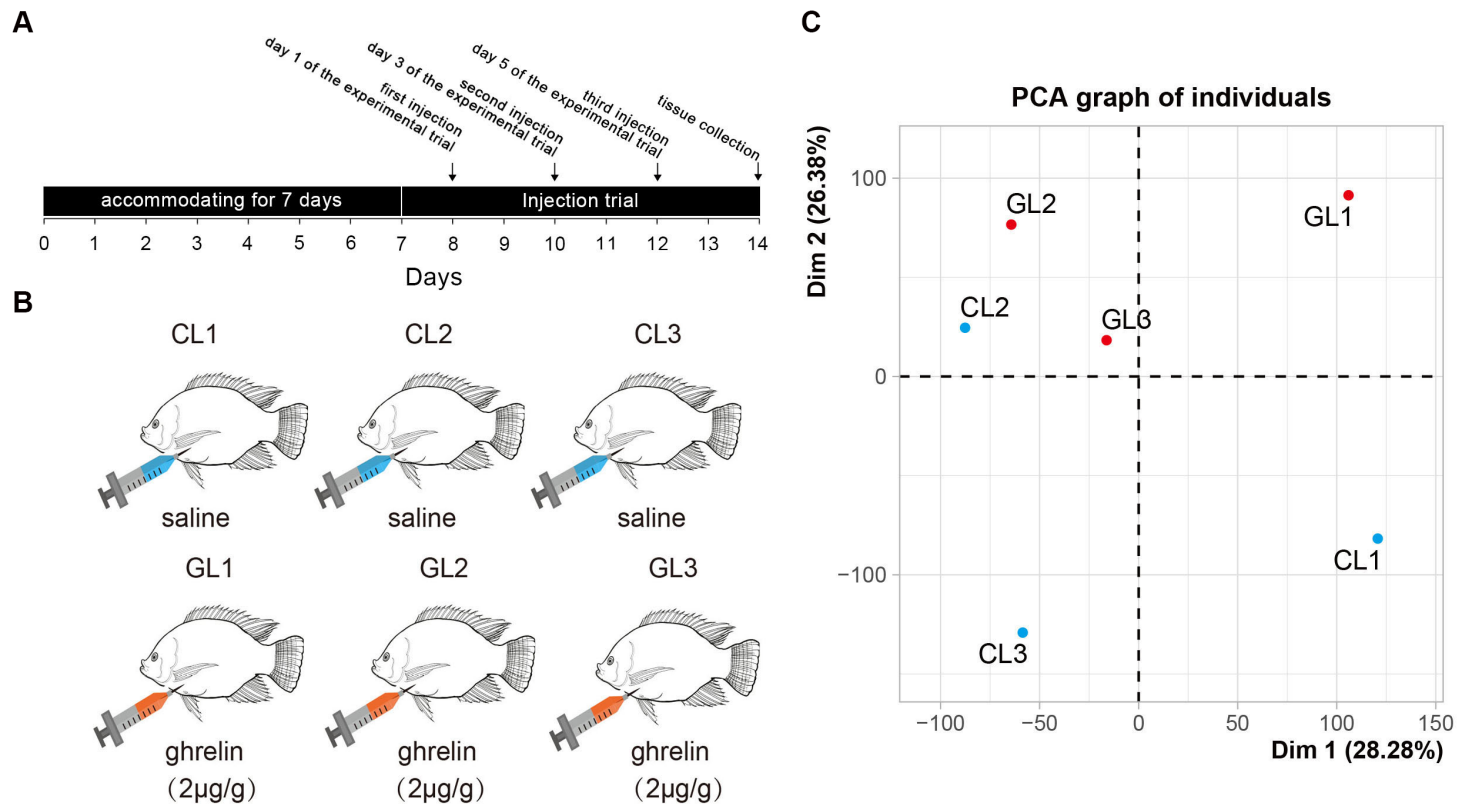
Experimental design and principal component analysis of transcriptome in the tested samples. **(A)** The scheme of experimental trial including time points of injection and sample collection. **(B)** Setting of experimental groups (ghrelin-injection, GL) and control (saline-injection, CL). **(C)** Results of principal component analysis of liver transcriptome in the two groups.

Nile tilapia for the experiments were sourced from the local market (Changsha, China). Before injection, we randomly selected 10 male fish (body weight: 0.40 ± 0.15 kg; full length: 25.23 ± 3.09 cm) and divided them into two groups: control (CL) and ghrelin-injected (GL) tilapia. The fish were allowed to accommodate for 7 days in the pool before the injection. Fish were cultured by group in two separate tanks (1 m × 1 m × 1.5 m) equipped with continuous flow systems at 25–28°C and PH 7.2, with a 12:12 h light: dark photoperiod. The mature peptide of tilapia was synthesized by GenScript Biotechnology (Nanjing, China) and the peptide sequence was CGSSFLSPSQKPQNKVKSSRIGR according to the sequence from NCBI GenBank (XP_025763099.1). In addition, we found that intraperitoneal injections of 2 μg/g body weight stimulated food intake in grass carp (unpublished data). Hence, we used 2 μg/g body weight of ghrelin for injection in the study. On day 1, day 3, and day 5 of the experimental trial (Figure 1A), we treated the fish in the CL group with saline (0.6 NaCl) and fish in the GL group with ghrelin (2 μg/g body weight, prepared in saline). The fish were fed with commercial diet (provided by Yuehai Feed Co., Ltd., China) twice a day (8:00 and 17:00).

The fish were first anesthetized by 60 mg/L MS-222 (CAS No.: 886–86-2, Sigma, United States). Subsequently, the livers of fish were obtained on day 7 and RNA-seq was implemented using three random samples in each group (Figure 1B). The liver tissues were excised out by sterile scissor and forceps. All the experiments have been approved by Ethics Committees of Hunan Normal University (Changsha, China).

### 2.2. RNA extraction and quality evaluation

Total RNAs of the liver samples were extracted with TRIzol reagent (CAS No.: 9109, Takara, Japan) following the manufacturer’s instructions. RNA integrity was checked by 1% agarose gel electrophoresis and the concentration was assayed by NanoDrop 2000 spectrophotometer (Thermo Scientific, United States). Aligent 2100 (Aligent, United States) was used to evaluate RNA quality.

### 2.3. cDNA library construction and RNA-seq

The mRNA was first isolated by oligo (dT)-attached magnetic beads. Subsequently, 100–500 bp fragments were obtained using a fragmentation buffer. These fragments were used as a template to synthesize cDNA with the NEB Next Ultra RNA Library Prep Kit (CAS No.: E7420, Illumina, United States) and random hexamer primers. PCR and agarose gel electrophoresis were performed sequentially to amplify and purify cDNA. Finally, cDNA libraries were sequenced on the Illumina HiSeqTM 2000 platform (Illumina, United States).

### 2.4. Read processing and function annotation

We obtained the clean reads by removing adaptor sequences and low-quality reads from the raw reads using in-house Perl scripts. Then, the clean reads were mapped to the Nile tilapia genome^1^ using RSEM software (14). To understand the gene functions, GO and KEGG analyses were performed to obtain biological information in relation to molecular function, cellular components, biological processes, and pathways relevant for the genes.

### 2.5. Identification of DEGs

Fragments per kilobase of exon model per million mapped reads (FPKM) was employed as the key indicator of gene expression levels. R package DESeq was used to identify the DEGs (15). DEGs between CL and GL were defined as those with an adjusted *p* ≤ 0.05 and |log2 fold change| ≥ 1.

### 2.6. GO and KEGG enrichment analysis

Differentially expressed genes were analyzed by GO enrichment using GOseq R package, and GO terms with an adjusted *p* ≤ 0.05 were considered enriched (16). Pathway enrichment was examined using KOBAS software (KOBAS, Surrey, United Kingdom) to identify enriched KEGG pathways (adjusted *p* ≤ 0.05) (17).

### 2.7. Real-time quantitative PCR

Using the PrimeScript™ RT reagent kit (CAS No.: RR037B, Takara), reverse transcription was performed to transform RNAs into cDNA with high quality and at high quantity. Subsequently, DNA Eraser (Takara) was used to eliminate the genomic DNA in RNA, and then β-actin primers were used as a house gene to evaluate cDNA quality.

The specific DEG primers were designed by Primer Premier 5.0 (Premier Biosoft International, USA; Supplementary Table S1). The cDNA product was subjected to qPCR on the QuantStudio™ 3 Real-Time PCR System (CAS No.: A28136, ABI, United States). Each reaction system included 1 μL cDNA template, 0.5 μL forward primer, 0.5 μL reverse primer, 5 μL fluorescent dye (PowerTrack Green Master Mix, CAS No.: A46112, ABI, United States), and 3 μL ddH_2_O. The reaction was performed under the following conditions: 50°C for 2 min, 95°C for 10 min, followed by 40 amplification cycles (95°C for 15 s and 60°C for 45 s). After amplification, the specificity of PCR was detected using the melting curve, and relative expression was calculated by 2^−ΔΔCt^ method (18). Finally, *t*-test was performed to test for significant differences between genes in the CL and GL groups in SPSS v17.0 (SPSS, Chicago, IL, United States).

## 3. Results

### 3.1. Analysis of RNA-seq

RNA-seq was performed on the livers of Nile tilapia in the CL and GL groups with three parallel samples in each group (CL1, CL2, CL3, GL1, GL2, and GL3). A total of 310,528,970 raw reads were generated. The largest sample among these was CL1 with 59,058,792 raw reads, and the smallest was GL2 with 41,924,288 raw reads. After removing low-quality reads, a total of 308,508,816 clean reads were obtained. Each sample had 51,418,136 clean reads on average (Supplementary Table S2). All the reads are available on NCBI SRA database.^2^ Among the six samples, the proportion of clean reads successfully mapped with the genome ranged from 91.8 to 93.5% (Supplementary Table S3). Moreover, principal component analysis (PCA) showed that all samples were clearly divided into two regions, with the GL samples concentrated in one region and the CL samples in the other region (Figure 1C), indicating differences in the expression of transcriptomes between the two groups.

### 3.2. GO and KEGG enrichment analysis of DEGs

**Figure 2:**
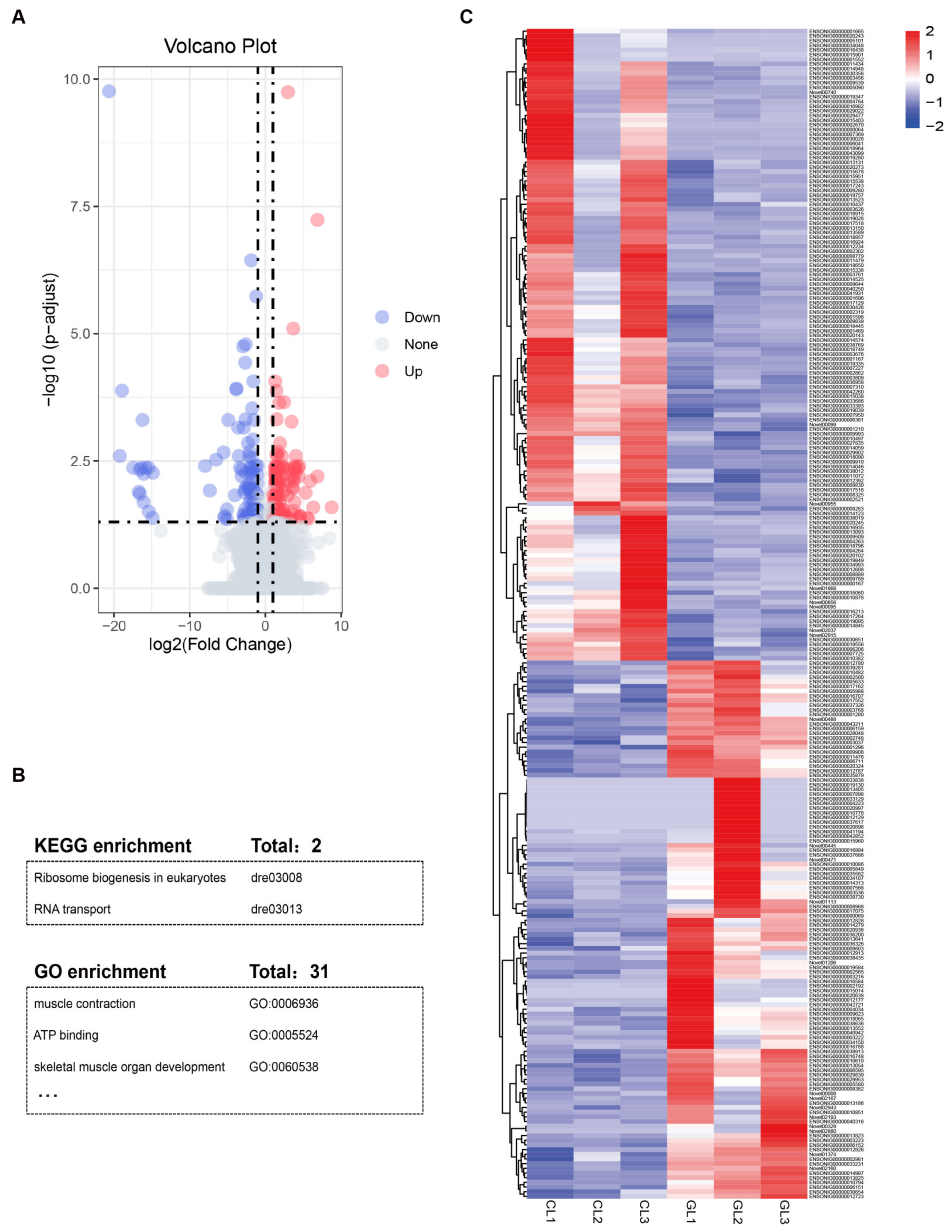
Summary of differentially expressed genes and function annotation. **(A)** Volcano plot of mRNAs expression in the GL vs. CL group. **(B)** Table of enriched KEGG pathways and GO terms. **(C)** Heatmap of the DEGs identified in RNA-seq data in the two groups.

Using the DESeq package, we identified a total of 250 DEGs (Supplementary Table S4). Among them, 135 genes were upregulated, namely, these genes had higher expression in the CL group than in the GL group. The remaining 115 DEGs were downregulated (Figures 2A,C). GO and KEGG enrichment analyses indicated that DEGs were significantly enriched in terms and pathways related to energy metabolism and RNA transcription (Figure 2B; Supplementary Tables S5, S6). ATP binding (GO:0005524) and muscle contraction (GO:0006936) are related to active energy metabolism functions of liver. Ribosome biogenesis in eukaryotes (dre03008) and RNA transport (dre03013) are associated with RNA transcription.

### 3.3. Ghrelin injection changes genes related to energy metabolism and RNA transcription

**Figure 3:**
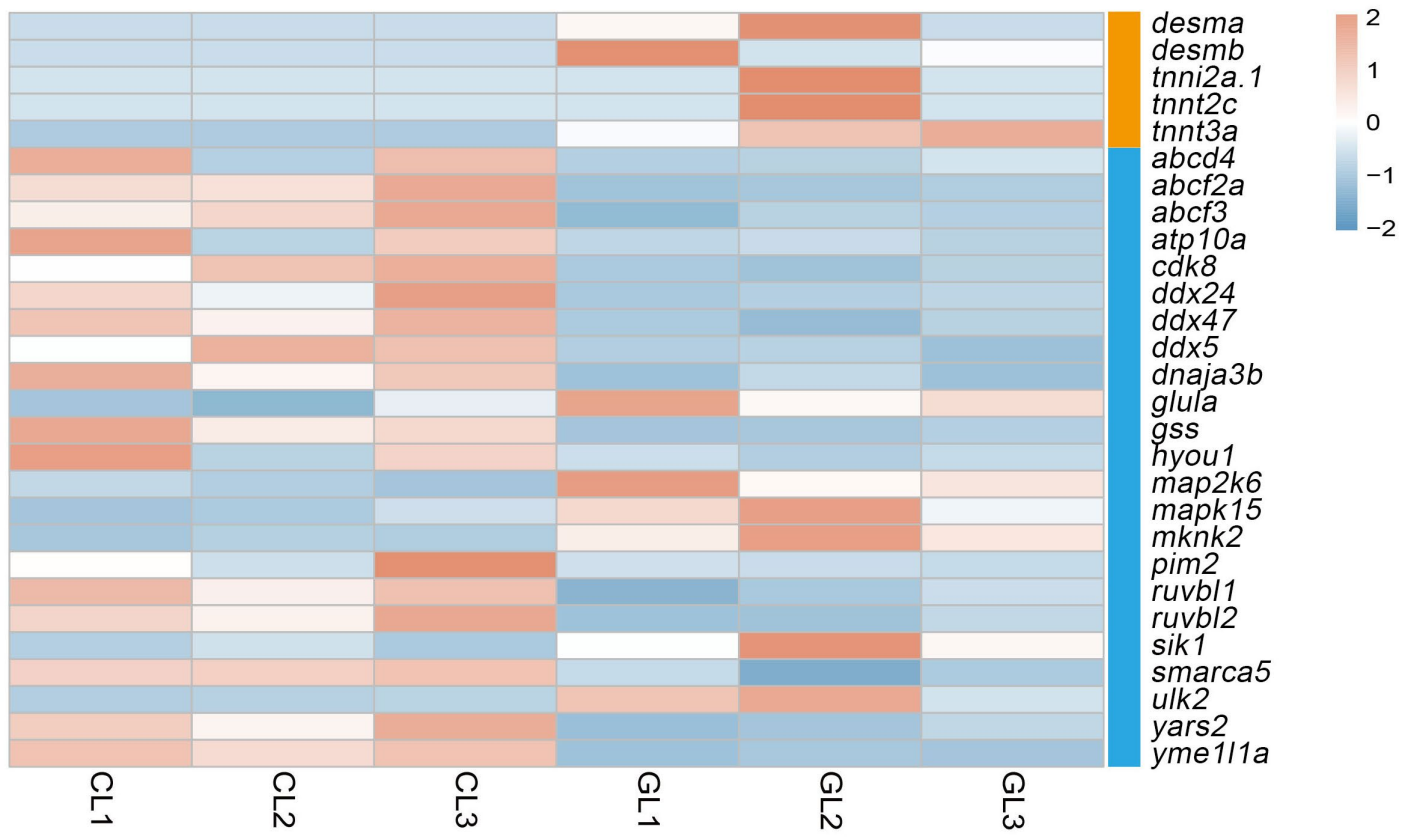
Heatmap of DEGs related to ATP-binding and muscle contraction terms. Orange denotes genes associated with the muscle contraction term, and blue denotes genes related to the ATP-binding term.

Liver performs metabolic functions, which require a large amount of energy; therefore, the supply of ATP is crucial. In this study, we focused on the ATP-binding and muscle contraction terms. Five genes related to muscle contraction were differently expressed. They encode desmin and troponin, and all of them were upregulated in the GL group. As for ATP binding, there were 23 DEGs in total. Among them, genes that encode the ATP-binding-cassette (ABC) system, DEAD box helicase (DDX), RuvB-like AAA ATPase (RUVBL) were downregulated in the GL group. Moreover, genes that encode the mitogen-activated protein kinase (*mapk*) and mitogen-activated protein kinase kinase (*map2k*) were upregulated in the CL group (Figure 3).

**Figure 4:**
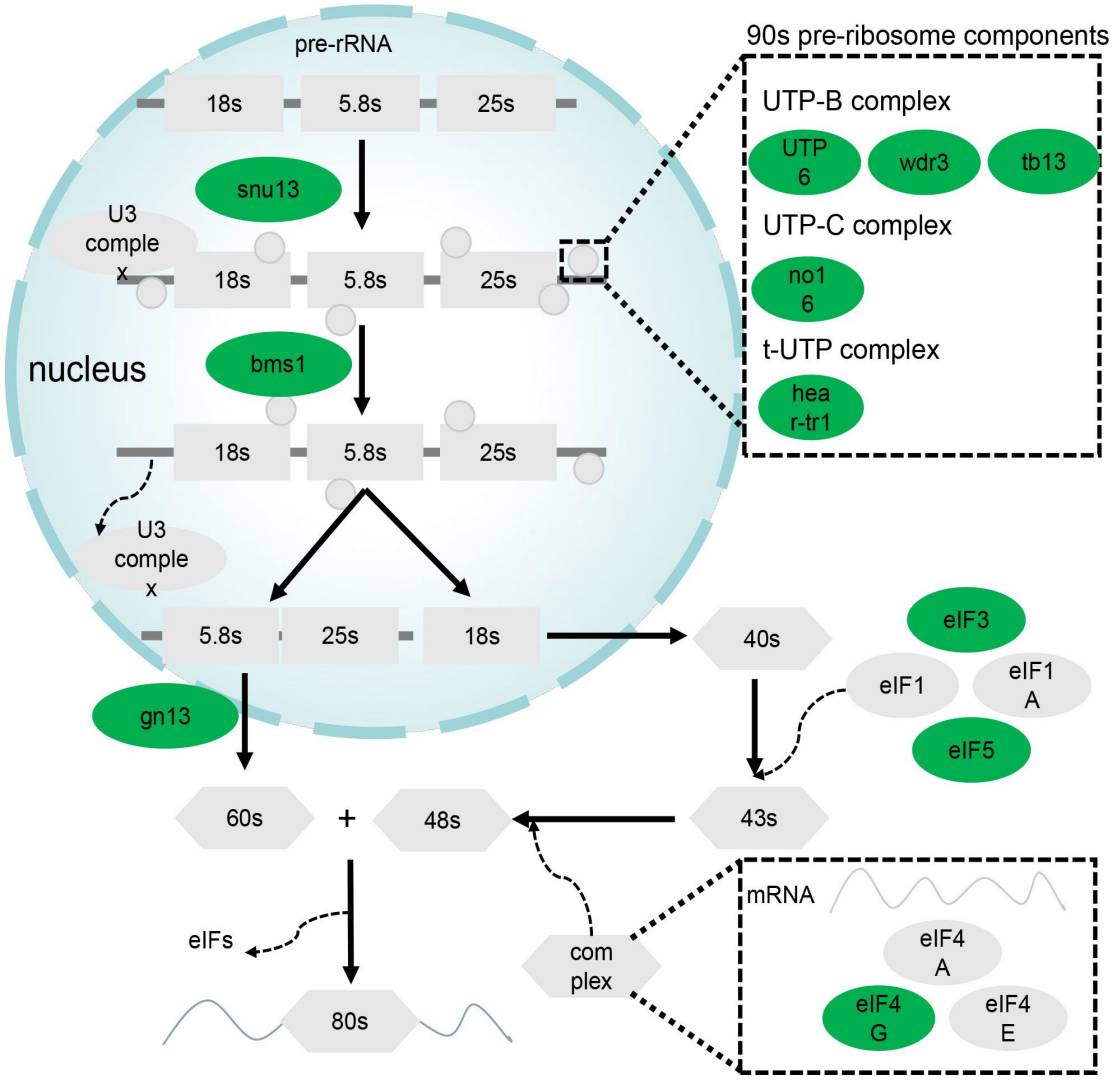
DEGs related to ribosome biogenesis in eukaryotes and RNA transport pathways. Green denotes genes downregulated in the GL group.

Ribosome biogenesis and RNA transport pathways are important for the role of liver in synthesis. A total of 14 genes in these pathways were differentially expressed; specifically, they were downregulated. These DEGs are mainly associated with 90s pre-ribosome components and eukaryotic initiation factor (*EIF*; Figure 4).

### 3.4. RT-qPCR of genes affected by ghrelin injection in liver

**Figure 5:**
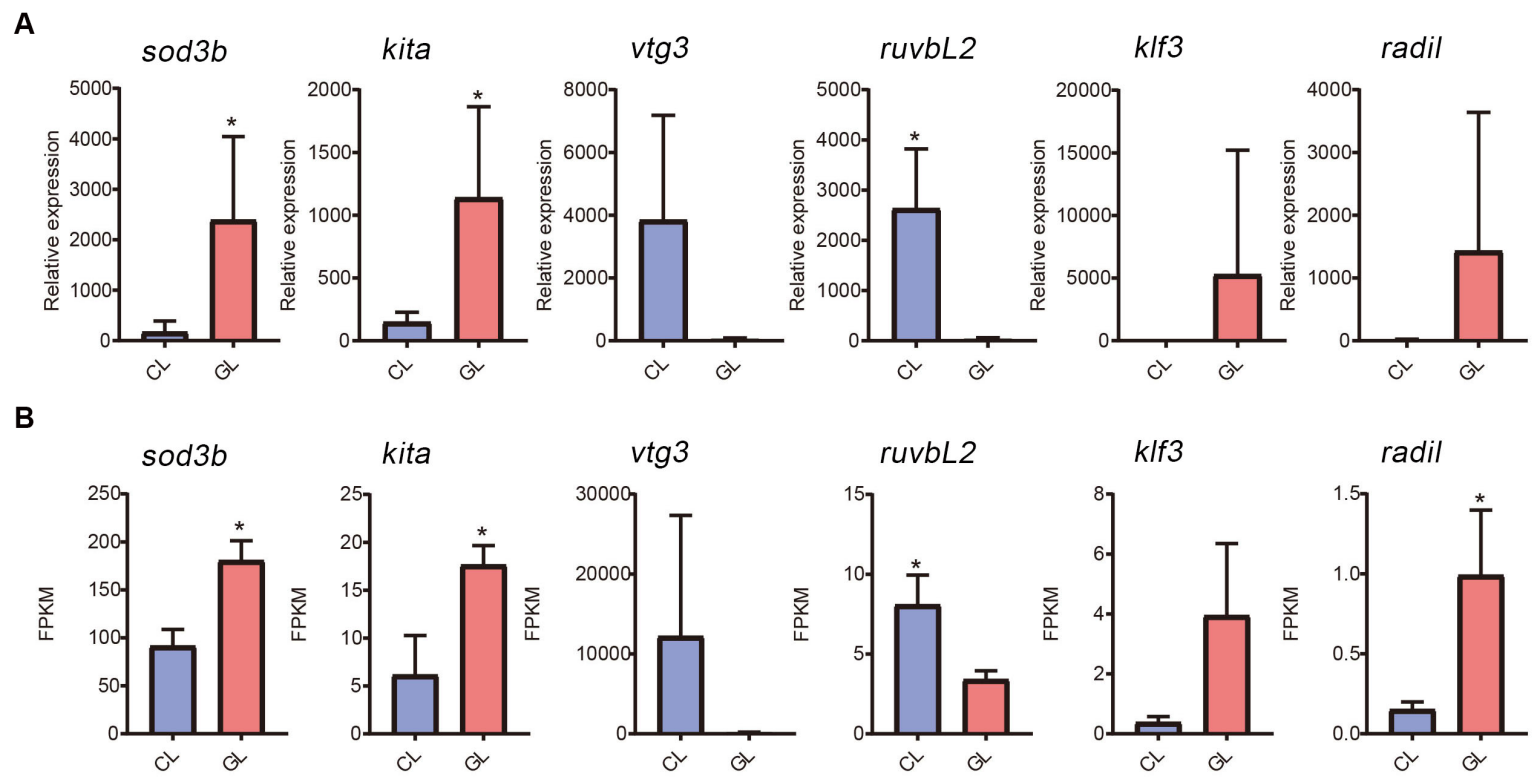
Comparison of gene expression between the transcriptome and RT-qPCR data. **(A)** Histogram of RT-qPCR results of relative expression. **(B)** Histogram of RNA-seq results of FPKM.

Real-time quantitative PCR was performed to validate gene expression detected by RNA-seq. Six genes were randomly selected (*sod3b*, *kita*, *vtg3*, *ruvbL2*, *klf3*, and *radil*). Among them, expression of *sod3b*, *kita*, *klf3*, and *radil* were higher in the GL group than in the CL group, while *vtg3* and *ruvbL2* were downregulated in the GL group (Figure 5A). The RNA-seq results were similar (Figure 5B). From the correlation between RNA-seq and RT-qPCR, the accuracy of RNA-seq was demonstrated.

## 4. Discussion

Ghrelin is a pleiotropic hormone. This study was designed to ascertain the effect of ghrelin on tilapia by analyzing the transcriptome changes in tilapia liver after ghrelin injection. We demonstrated that after ghrelin injection, the metabolic activity of liver was altered, which mainly manifested in changes of gene expression related to ATP binding, muscle contraction, ribosome biogenesis in eukaryotes, and RNA transport. Changes in the expression of these important genes contribute to our understanding of ghrelin at the molecular level and provide an option to promote the growth of tilapia.

ATP binding and muscle contraction are related to energy metabolism and were used to evaluate liver function. Genes encoding *desmin* and *troponin*, which are related to muscle contraction, were upregulated in the GL group. Both of these proteins are important components of the cytoskeleton (19). A previous study has shown that loss of *desmin* resulted in a decrease in mitochondria and their impaired function, which linked *desmin* to respiration (20). The gene *troponin* can activate myosin ATPase to contract muscles, indicating a correlation between troponin and energy consumption (21). High expression of *desmin*- and *troponin*-encoding genes in the GL group indicated a positive effect of ghrelin on the development and maintenance of muscle in tilapia and high energy metabolism. A total of 23 DEGs related to ATP binding were identified. Among them, DEGs that encoded the ABC system, DDX, and RUVBL were all downregulated in the GL group. In addition to ATPase activity, which can consume ATP to transport substances, the ABX family also participates in other processes such as signal transduction (22). A previous study found that the expression of genes encoding the ABC system was downregulated after ghrelin injection in mice, thus reducing the transfer of abdominal fat and leading to fat accumulation through the action of ghrelin on GHS-R (23). Our results were consistent with these findings, providing evidence of the role of ghrelin in growth promotion. DDX and RUVBL belong to ATP-dependent RNA and ATP-dependent DNA helicases, respectively, which regulate gene expression (24). Surprisingly, although ghrelin has a growth-promoting effect, the expression of genes related to growth and development was attenuated after ghrelin injection. *Mapk* and *map2k* are related to the MAPK signaling pathway, which regulates many cellular processes such as transcription activation and apoptosis (25). High expression of *mapk15* and *map2k6* in the GL group may indicate high cellular physiological activity. In zebrafish, IGF-1 stimulated the expression of the MAPK signaling pathway to promote cell proliferation (26). The activation of the MAPK signaling pathway by ghrelin may be mediated through IGF-1. Therefore, the effects of ghrelin on cellular energy utilization and regulation of physiological activities were not clear and we were unable to determine the direction of the effect (inhibition or promotion).

Ribosome biogenesis in eukaryotes and RNA transport are related to the efficiency of translation, which can determine the amount of protein synthesized. The DEGs related to ribosome biogenesis in eukaryotes were all downregulated in the GL group. As these genes contribute to ribosome formation, a decrease in ribosome synthesis in the GL group can be inferred. Several gene including *eif4g1a*, *eif3c*, *eif5b*, *trnt1*, *prmt5*, and *nup155* were the DEGs related to RNA transport and their expression was lower in the GL group. Among them, *eif4g1a*, *eif3c*, and *eif5b* encode the eukaryotic initiation factor (eIF). It has been reported that eIF interacts with mRNA, ribosomes, and other factors to complete the translation process (27). Downregulation of eIF would slow down translation and cell growth. In summary, we found that the translation efficiency of the liver slowed down after ghrelin injection, which was not expected based on current understanding.

Ghrelin is a pleiotropic hormone, which is involved in many pathways and participates in a variety of physiological activities. Ghrelin can influence the liver through a variety of ways such as the ghrelin-GHS-R and GH/IGF-1 axes and its effect on AMPK. In this study, ghrelin likely influenced the changes in energy metabolism in the liver through the ghrelin-GHS-R and GH/IGF-1 axes, as key DEGs related to ATP binding and muscle contraction are closely associated with GHS-R and IGF-1. However, the reasons for the downregulation of genes related to ribosome biogenesis in eukaryotes and RNA transport in response to ghrelin injection are not clear and may be based on a more complex mechanism.

In this study, we analyzed changes in the transcriptome of tilapia liver after ghrelin injection. In summary, energy metabolism and substance synthesis in the liver were altered after ghrelin injection, as indicated by the changes in the expression of genes related to ATP binding, muscle contraction, ribosome biogenesis in eukaryotes, and RNA transport. This result provides a new perspective for understanding the function of ghrelin on the liver and provides new information for the promotion of growth in tilapia.

## Data availability statement

The datasets presented in this study can be found in online repositories. The names of the repository/repositories and accession number(s) can be found at: PRJNA856826 (NCBI-Bioproject-SRA).

## Ethics statement

The animal study was reviewed and approved by The Ethics Committees of Hunan Normal University.

## Author contributions

HZ and YZ designed the experiment and reviewed and revised the manuscript. CL and BR wrote the manuscript. JP and TD performed the data analysis. WQ performed the experiment. All authors contributed to the article and approved the submitted version.

## Funding

This study was supported by the National Key R&D Program of China (2022YFD2400102), the National Natural Science Foundation of China (31760756), the Hunan Provincial Natural Science Foundation of China (2021JJ20033 and 2022JJ30386), and the State Key Laboratory of Developmental Biology of Freshwater Fish (2021KF006).

## Conflict of interest

The authors declare that the research was conducted in the absence of any commercial or financial relationships that could be construed as a potential conflict of interest.

## Publisher’s note

All claims expressed in this article are solely those of the authors and do not necessarily represent those of their affiliated organizations, or those of the publisher, the editors and the reviewers. Any product that may be evaluated in this article, or claim that may be made by its manufacturer, is not guaranteed or endorsed by the publisher.

## Footnotes

^1^ https://asia.ensembl.org/Oreochromis_niloticus/Info/Index

^2^ https://www.ncbi.nlm.nih.gov/bioproject/?term=PRJNA856826
